# Changes in the hydro-climatic regime of the Hunza Basin in the Upper Indus under CMIP6 climate change projections

**DOI:** 10.1038/s41598-022-25673-6

**Published:** 2022-12-12

**Authors:** Aftab Nazeer, Shreedhar Maskey, Thomas Skaugen, Michael E. McClain

**Affiliations:** 1 Department of Water Resources and Ecosystems, IHE Delft Institute for Water Education, P.O. Box 3015, 2601 DA Delft, The Netherlands; 2 Department of Water Management, Delft University of Technology, P.O. Box 5048, 2600 GA Delft, The Netherlands; 3 Department of Agricultural Engineering, Bahauddin Zakariya University (BZU), P.O. Box 60800, Multan, Pakistan; 4 Norwegian Water Resources and Energy Directorate, P.O. Box 5091, Maj. 0301 Oslo, Norway

**Keywords:** Climate change, Hydrology

## Abstract

The Upper Indus Basin (UIB) heavily depends on its frozen water resources, and an accelerated melt due to the projected climate change may significantly alter future water availability. The future hydro-climatic regime and water availability of the Hunza basin (a sub-basin of UIB) were analysed using the newly released Coupled Model Intercomparison Project Phase 6 (CMIP6) climate projections. A data and parameter parsimonious precipitation-runoff model, the Distance Distribution Dynamics (DDD) model, was used with energy balance-based subroutines for snowmelt, glacier melt and evapotranspiration. The DDD model was set up for baseline (1991–2010), mid-century (2041–2060) and end-century (2081–2100) climates projections from two global circulation models (GCM), namely EC-Earth3 and MPI-ESM. The projections indicate a substantial increase in temperature (1.1–8.6 °C) and precipitation (12–32%) throughout the twenty-first century. The simulations show the future flow increase between 23–126% and the future glacier melt increase between 30–265%, depending on the scenarios and GCMs used. Moreover, the simulations suggest an increasing glacier melt contribution from all elevations with a significant increase from the higher elevations. The findings provide a basis for planning and modifying reservoir operation strategies with respect to hydropower generation, irrigation withdrawals, flood control, and drought management.

## Introduction

Climate change (CC) is accelerating (IPCC 2021^1^). Due to the continuous emission of greenhouse gases and subsequent warming, the threat of CC is continuously rising^2^. It was urged in the “2015—Paris Agreement” to limit the global temperature rise below 2 °C relative to the pre-industrial (1861–1890) level and stabilise it to 1.5 °C by 2100^3^. However, such an ambitious goal requires extraordinary efforts to increase the current levels of nationally determined contributions (NDCs) by 3–5 times^4^. The recent CC acceleration heightens concerns about future water availability from the high-altitude basins^5^. CC will change the frequency and magnitude of climatic variables such as precipitation and temperature^6,7^. Such changes are likely to be prominent in the Asian, South American and European low-latitude regions, where alpine glaciers are particularly sensitive to the prevailing climatic warming^8^. The Hindukush Karakoram Himalaya (HKH) is a region where the problem of vanishing glaciers is critical and will affect water availability in the next few decades^9^. The high-altitude HKH region and Indus basin are recognised as a “hotspot” of CC due to significant transformations in the hydro-climatic regime^10^. However, an accurate assessment of CC and associated impacts on hydrological regimes in the region is difficult due to limited data and insufficient analyses^3^.

Recent studies (1979–2010) of the winter westerlies, the primary source of precipitation in the Karakoram, indicated an enhanced frequency and increased amount of winter precipitation^11^. The Upper Indus Basin (UIB) showed significantly increased annual and seasonal precipitation at several stations from 1961 to 1990^12^. Also, consistent with the global trends, increasing temperature trends are evident for UIB. Lalande et al.^13^ analysed the near-surface air temperature, snow cover extent and precipitation over High Mountain Asia (HMA) using Coupled Model Intercomparison Project phase 6 (CMIP6) projections. Their analysis indicated an increased temperature, decreased snow cover extent and increased precipitation by the end of the century. Abbas et al.^14^ evaluated the performance of CMIP6 based general climate models (GCM) for precipitation projections over Pakistan. Shafeeque and Luo^15^ proposed a multi-perspective approach to select the best-suited GCMs to simulate the glacio-hydrology of UIB. Their analysis predicted a decrease in the area, volume, and length of selected glaciers. Akhtar et al.^16^ used the Special Report on Emissions Scenarios (SRES) data for UIB’s climatic modelling from 2071 to 2100. They predicted a mean temperature rise of 4.8 °C and a mean precipitation increase of 16% by the end of the twenty-first century. Sharif et al.^17^ evaluated the air temperature trends for UIB. They concluded that (i) the daily temperature range is consistently widening for all seasons, (ii) mean and maximum temperatures of winter show significant increases and (iii) mean and minimum summer temperatures show a decreasing trend. Negative temperature trends for summer from 1958 to 1990 and a positive trend from 1991 to 2001 were found for the Baltoro glacier in the Karakoram using the ERA-40 reanalysis dataset^18^. These future precipitation and temperature projections are quite uncertain and need further investigation.

The lack of observations, complex topography and interactions with synoptic-scale climatic circulations pose significant uncertainties in precisely representing the Indus basin’s hydro-climatic regime^3,7^. Consequently, conflicting trends have been observed in UIB regarding the CC impacts on the glaciers, and a debate has been prevailing concerning these trends during the last decade^7^. Hewitt^19^ observed glacier expansion and advancement in the central Karakoram. Sharif et al.^17^ and Tahir et al.^20^ indicated that large parts of the UIB are not yet experiencing accelerated melt, possibly due to the Karakoram anomaly. A more recent study in the central Karakoram (Shigar river basin) reported an increased flow^21^. Lutz et al.^7^ concluded that glacial melt contribution increases with neutral mass balance, with temperature and precipitation increase. However, future climatic projections are subjected to variabilities and large spread in the GCM. The GCMs are consistent for temperature projections with slight variation, whereas the precipitation projections vary highly, ranging from significantly drier to moderately wetter trends^7^. The projected global warming of 1.5 and 2 °C could increase the river flow by 34 and 43% from the upper Indus basin, according to Hasson et al.^6^. Hence, significant and accelerated changes are expected for the basin’s hydro-climatic regime^3^.

The recent acceleration in glacier melt due to CC in HKH mountains poses serious concerns about the glacier’s contribution to South Asian rivers^22^. It further illustrates that scientists and experts understand very little about these processes^22,23^; hence, precise simulations of these changes are lacking. About 70% of annual flow in the Indus River comes from glaciers and seasonal snowmelt^24^, so changes will directly affect the Indus flow and, consequently, millions of people downstream^20^. Pakistan is an agro-based country with 70% of its population dependent (directly or indirectly) on agriculture, so the water flows from the mountain headwaters are crucial. The Hunza basin in the western Karakoram is highly vulnerable to prevailing and future CC since 31% of its area is covered by glaciers (RGI, V6.0^25^). The Hunza’s flow depends on meltwater and the monsoon rain, which is available for a few summer months^26^. This water is stored in the country’s largest reservoir, Tarbela Dam, for irrigation needs and hydropower production throughout the year. Therefore, modelling the melt contribution to the river flow under CC scenarios is essential. This modelling may assist in managing the current and future domestic water supply, flood mitigation and hydropower productions^27^.

The current study attempts to simulate the hydro-climatic regimes based on CC projections and over the Hunza basin of UIB. It analyses the recently released projections by CMIP6^28^. The CMIP6 projections differ from CMIP5 with a new generation of climate models and a new set of periods, emissions, and land-use scenarios^29^. Moreover, to our knowledge, none of the studies has analysed the CMIP6 projections for the Hunza basin. These newly released precipitations and temperature projections are used as input to a precipitation-runoff model, the Distance Distribution Dynamics (DDD) model, where energy balance approaches are used to simulate snowmelt, glacier melt and evapotranspiration.

## Results

### Flow simulations for the baseline period

**Figure 1:**
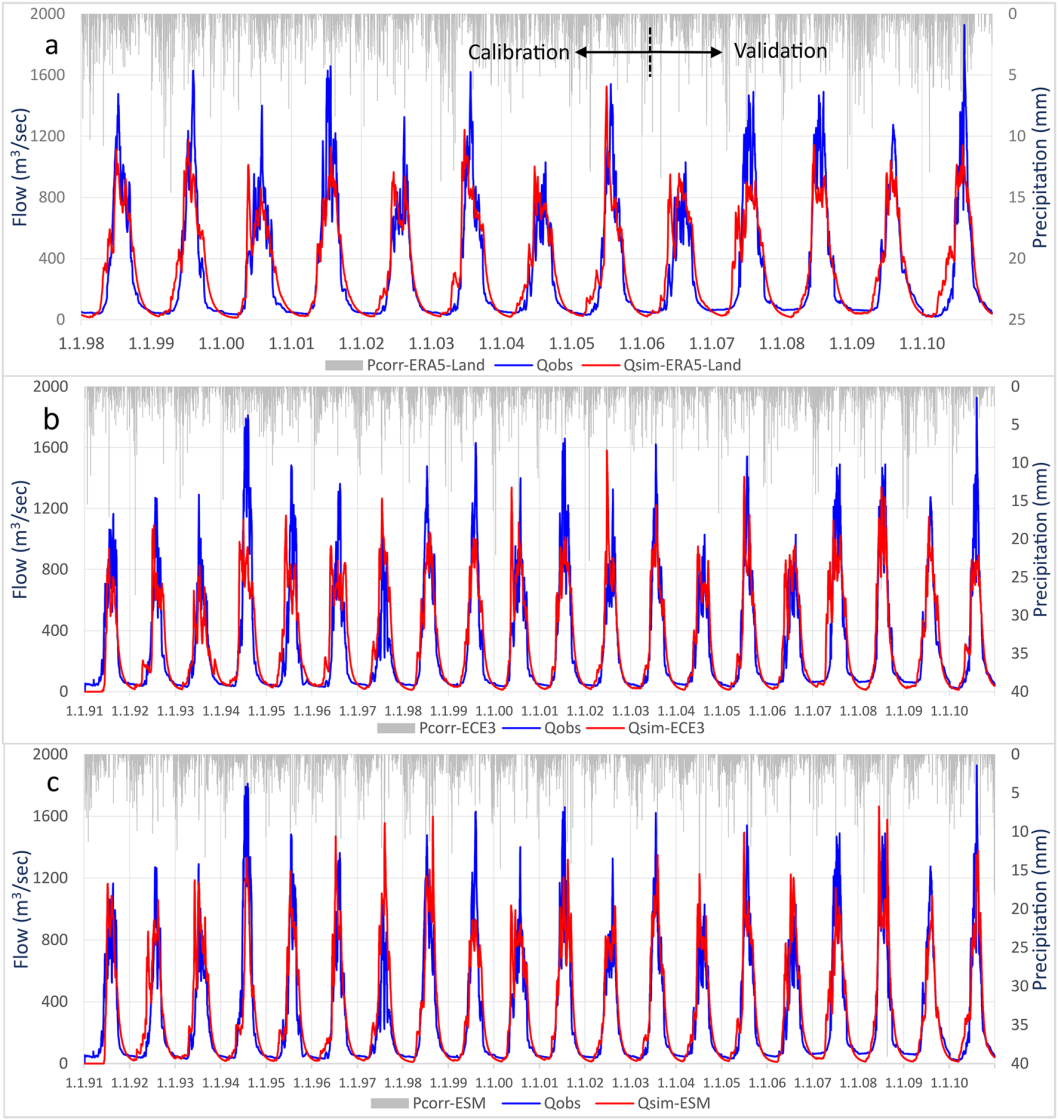
Observed vs simulated flow by DDD model for; (**a**) ERA5-Land based inputs (1998–2010), (**b**) ECE3 GCM based inputs (1991–2010), and (**c**) ESM GCM based inputs (1991–2010).

Figure 1A shows the European Reanalysis 5 Land (ERA5-Land) based daily simulated and observed flow for the calibration and validation period together with the corrected precipitation. The flow simulation showed reasonable results with performance efficiencies Kling Gupta Efficiency (KGE) of 0.82 and Nash–Sutcliffe Efficiency (NSE) of 0.80. These 1997–2010 simulations for the Hunza basin using ERA5-Land precipitation inputs are similar to those in Nazeer et al.^26^. However, the revised subroutine for simulating glacier melt changed the results slightly.

To validate the bias-corrected elevation-distributed GCM precipitation and temperature, simulations were performed for the baseline period for the Hunza basin. The time series of the daily simulated and observed flow for 1991–2010 and the bias-corrected precipitation are shown in Fig. 1b for European Earth consortium (hereafter called the ECE3) based inputs and Fig. 1c for Earth System Model (ESM). The simulated flow is in good agreement with the observed flow. The ECE3 based simulation had a slightly overestimated flow of 5.1%, and the ESM based simulation, 8.1%. The observed and simulated low flows and flow recessions for the baseline period are in good agreement. The mean monthly simulated flow is also in good agreement but with the slightly less simulated base flow. The high flow regime indicates significant flow from April to October, with peaks in July and August. The low flow period is from November to March, with the minimum flow in February and March.

### SCA, SWE and GM simulations for the baseline period

GCMs based simulated snow cover area (SCA) and GM for 1991–2010 for the Hunza basin are shown in Fig. S1. When the temperature rises in March, the snow starts melting, and the SCA decreases to its minimum in August. With the temperature decreasing in September, the precipitation falls as snow in most of the basin, and SCA starts increasing and peaks in February. Elevation-distributed means of simulated SCA, snow water equivalent (SWE) and glacier melt (GM) from GCMs compared with the ERA5-Land based simulations for the Hunza basin for 1991–2010 are shown in Table 1 and are in good agreement. The lowest elevations have the minimum SCA, while the higher elevations are mostly snow covered the whole year. The SWE follows the same melt and accumulation patterns as SCA for each elevation. SCA and SWE both increase from lower to higher elevations. SWE estimates are slightly higher with the GCM based simulations than the ERA5 based simulations.

**Table 1:** Elevation-distributed annual average snow cover area (SCA), snow water equivalent (SWE), and glacier melt (GM) for ERA5-Land and GCM based inputs for the baseline period.

Simulation	a1	a2	a3	a4	a5	a6	a7	a8	a9	a10	Mean
**SCA (%)**											
*ERA5-Land*	17	39	48	62	68	74	77	79	81	98	64.3
*ECE3-GCM*	16	34	47	62	68	74	76	79	82	100	63.7
*ESM-GCM*	14	35	48	64	69	75	76	78	80	97	63.6
**SWE (mm)**											
*ERA5-Land*	78	56	191	246	287	303	312	313	306	317	241
*ECE3-GCM*	57	172	214	253	285	291	290	289	301	430	258
*ESM-GCM*	56	177	222	262	295	302	303	302	313	450	268
**Glacier cover (%)**	1.6	4.7	5.4	6.0	6.8	8.1	10.2	13.4	17.2	26.7	100
**GM (mm)**											
*ERA5-Land*	28	66	62	52	48	47	53	64	71	12	502*
*ECE3-GCM*	29	67	65	54	51	50	56	66	73	1	512*
*ESM-GCM*	28	66	65	55	53	53	61	74	84	13	553*

*This glacier melt value is for the glacier area only, not for the whole basin.

The time series of GCMs based simulated GM for the Hunza basin from 1991 to 2010 is shown in Fig. S1. The glaciers contribute throughout the year, except for the winter months (Dec–Feb). However, the melt contribution is significant from May to September, with a peak in July/August. In the early summer, the glaciers at lower elevations start contributing and then a further increase in temperature in the late summer generates melt at higher elevations. The elevation-distributed GM (Table 1) indicated that glaciers from all elevations are melting and contributing significantly to the flow. The lower elevations start contributing very early in spring and keep contributing until the start of December.

### Temperature projections

**Figure 2:**
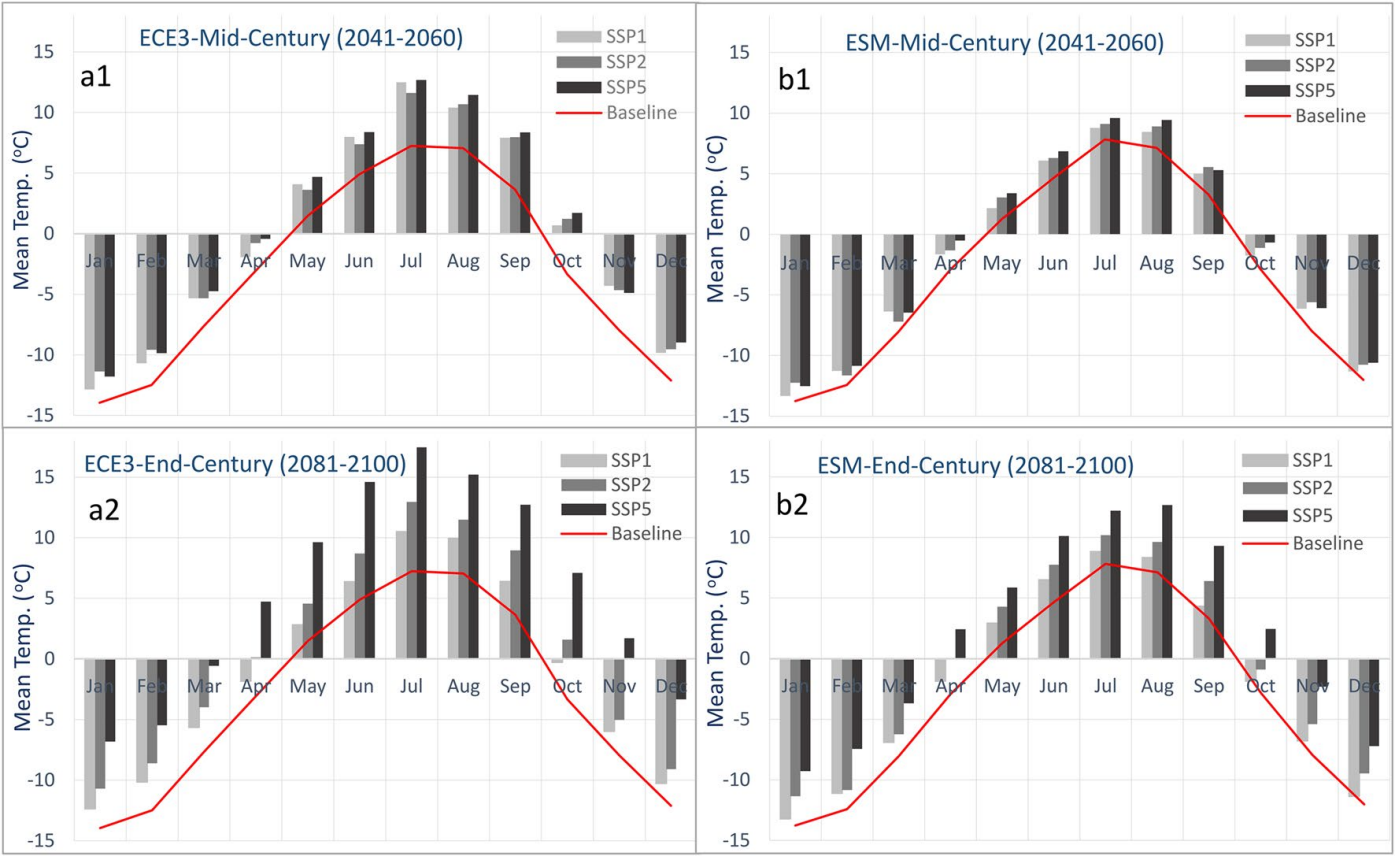
Mean monthly future temperature relative to the baseline period based on; (**a1**) ECE3-mid-century, (**a2**) ECE3-end-century, (**b1**) ESM-mid-century, and (**b2**) ESM-end-century.

Figure 2 shows the Hunza basin’s mean monthly temperature for baseline, mid-century and end-century periods based on ECE3 and ESM GCMs under Shared Socioeconomic Pathways (SSP) scenarios of SSP1, SSP2, and SSP3. A temperature increase is evident in all scenarios from the baseline period to mid- and end-century. The mean monthly basin baseline temperature is below 0 °C for October–April in both GCMs. The mean monthly basin-scale ECE3 temperature estimates remain below 0 °C for November–April for mid-century periods. The months with temperatures below 0 °C are reduced to December-March for end-century. The ESM estimates also show October and November with temperatures below 0 °C for mid- and end-century. Most severe warming is expected for the end-century SSP5 scenario. The minimum (monthly mean) increase from the baseline temperature to the future is 1.1 °C for December for SSP1-mid-century, and the maximum is 8.6 °C in July for SSP5-end-century based on ECE3. The warming differences are less in ESM, with a minimum of 0.5 °C for December for SSP1-mid-century and a maximum of 5.5 °C for SSP5-end-century for August. However, both GCMs indicate strong warming in July and August, the most intense glacier melt period.

The annual temperature increase is also evident from the baseline period to mid- and end-century for both GCM and all scenarios except SSP1. The SSP1 indicates an increase for mid-century but a temperature drop for the end-century period. The ECE3 GCM shows an increase in temperature for mid- and end-century scenarios compared to ESM.

### Precipitation projections

**Figure 3:**
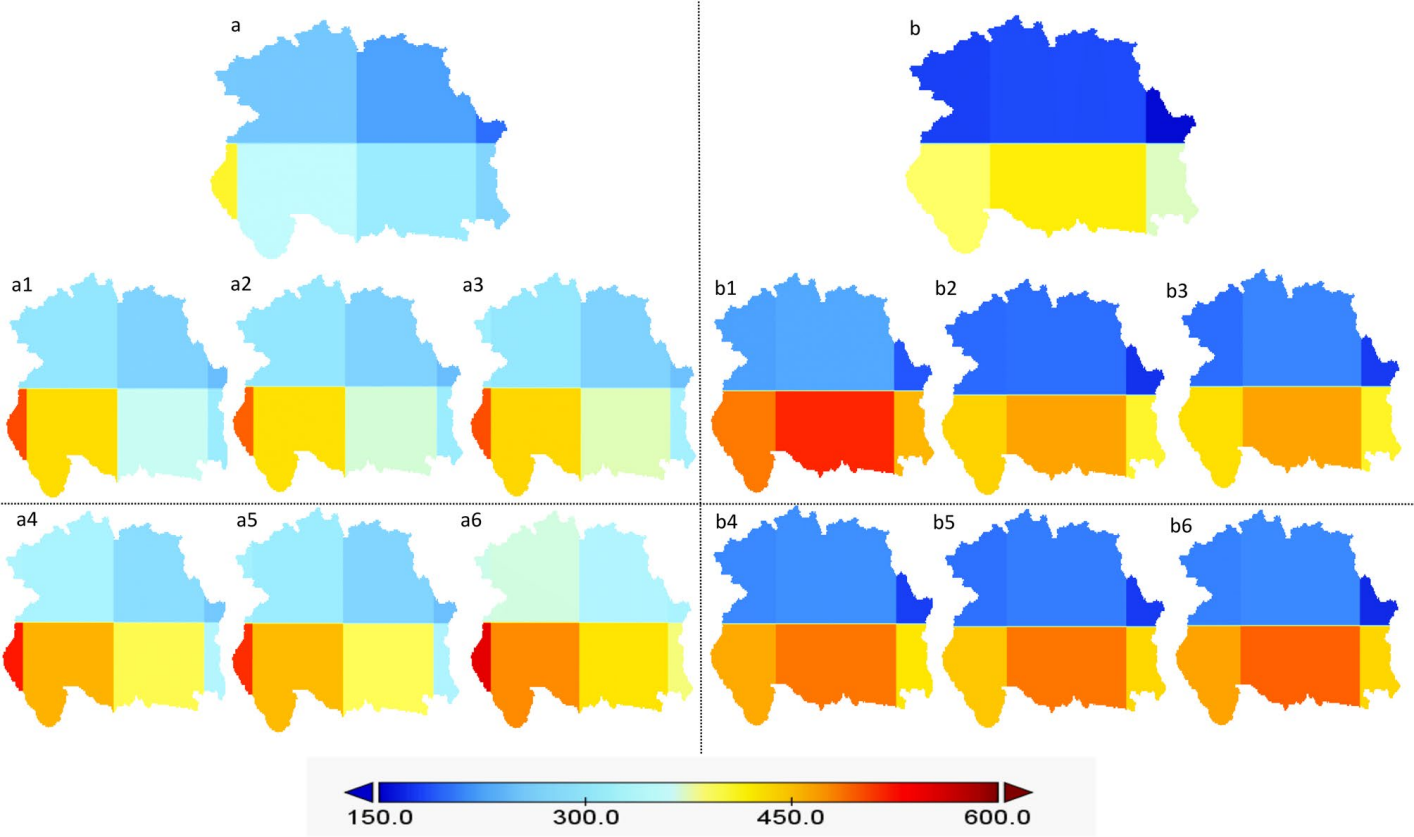
Mean annual basin spatial precipitation (mm) based on (**a**) ECE3-baseline, (**a1**) ECE3-SSP1-mid-century, (**a2**) ECE3-SSP2-mid-century, (**a3**) ECE3-SSP5-mid-century, (**a4**) ECE3-SSP1-end-century, (**a5**) ECE3-SSP2-end-century, (**a6**) ECE3-SSP5-end-century and (**b**) ESM-baseline, (**b1**) ESM-SSP1-mid-century, (**b2**) ESM-SSP2-mid-century, (**b3**) ESM-SSP5-mid-century, (**b4**) ESM-SSP1-end-century, (**b5**) ESM-SSP2-end-century, and (**b6**) ESM-SSP5-end-century.

The annual mean precipitation for baseline and future (mid-century and end-century) periods for all selected scenarios and GCMs are shown in Fig. 3. Relative to the baseline period, the ECE3 GCM shows 19–32% increases in annual precipitation and ESM shows 12–28% increases for the twenty-first century. Moreover, precipitation as snow reduces and rainfall increases. Maximum precipitation is in the winter/spring season, and minimum precipitation is in the post-monsoon season. The monthly estimates are similar for both GCMs. The monthly precipitation changes in GCM’s future projections relative to the baseline period are shown in Table S3.

### Flow simulations for the future period

**Figure 4:**
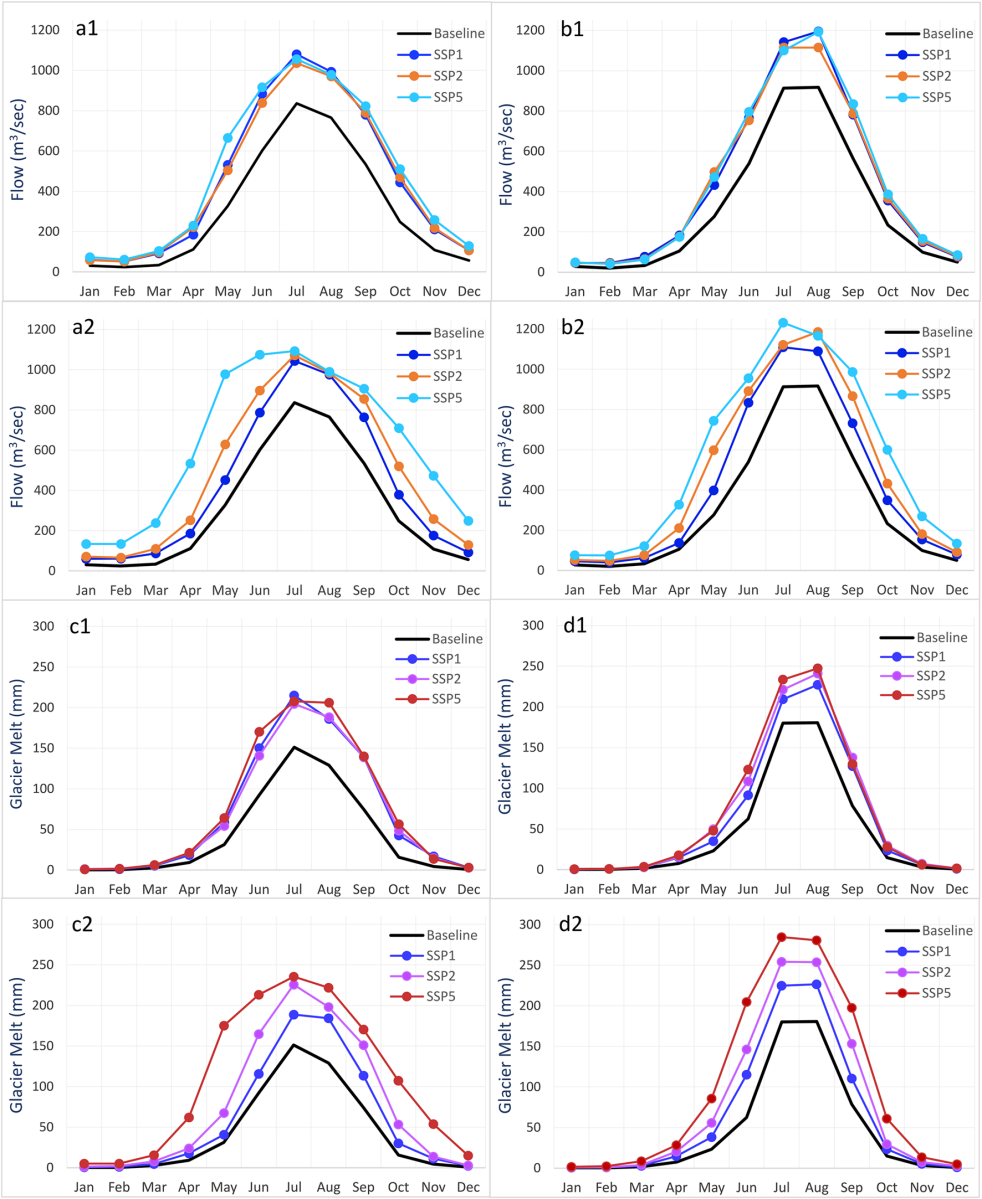
Mean monthly simulated future flow (Qsim) and glacier melt (GM) relative to the baseline; (**a1**) ECE3-Qsim for mid-century, (**a2**) ECE3-Qsim for end-century, (**b1**) ESM-Qsim for mid-century, (**b2**) ESM-Qsim for end-century, (**c1**) ECE3-GM based mid-century, (**c2**) ECE3-GM based end-century, (**d1**) ESM-GM based mid-century and (**d2**) ESM-GM based end-century.

The mean monthly simulated flow using ECE3 and ESM precipitation and temperature inputs for all scenarios is shown in Fig. 4. The flow increases from the baseline to the mid-century period and also from the mid-century to the end-century period. The baseline simulation for both GCMs showed a minimum mean monthly flow in February and a maximum in July. For future scenarios, the ECE3 based simulations have peak flow in July, but ESM has peak flow in August except for SSP5-end-century. In addition, ECE3 based flows are slightly higher than the ESM based simulations.

### Glacier melt simulations for the future period

The GM simulations driven by bias-corrected GCMs inputs are presented in this section. Figure 4 shows the mean monthly simulated GM with GCMs based precipitation and temperature inputs for all SSP scenarios. Relative to the baseline simulated GM, the future simulated GM is significantly higher for all selected scenarios. Against the baseline period annual glacier melt of 2193 Mm^3^ from the Hunza basin, the simulations show the melt volume increase between 3027 and 5813 Mm^3^ (38–265%) by the end of the twenty-first century. In addition, the peak melt period expands from July–September to May–October for future scenarios. Compared to the ESM based simulations, ECE3 based simulations produce slightly higher glacier melt for all scenarios and future periods.

The changes in elevation-distributed glacier melt for future periods relative to the baseline are shown in Table 2. The baseline value represents the elevation-distributed simulated GM using the baseline period data. There is a higher elevation-distributed glacier melt contribution for the future periods for all scenarios and GCMs. The GM differences from the baseline to the future are minimum for lower and maximum for higher elevations. For the baseline period, the highest elevation zone (a10) with the maximum glacier extent (about 27% of the total) contributed as little as 1–2% (annual mean) of total melt. For the future period, the contributions from the same elevation are 16–22% (annual mean) of total melt for SSP5-end-century simulations. The future glacier melt contribution will significantly increase from the higher elevations since about 68% of the glaciers are located in the upper half of the Hunza basin.

**Table 2:**
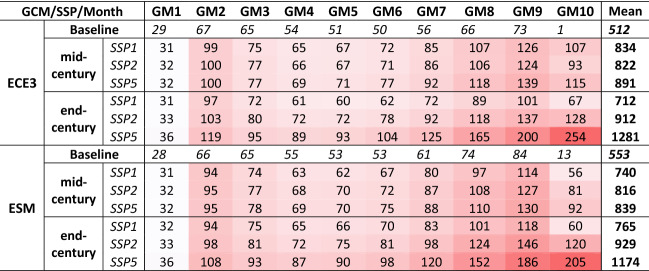
Elevation-distributed mean annual glacier melt (GM) in mm in the Hunza basin for baseline and future periods under all scenarios.

### SCA and SWE simulations for the future period

Table 3 shows changes in the percent of the future mean monthly SCA relative to the baseline of the ECE3 and ESM based simulations for all scenarios and periods. While Table 4 shows the same for SWE. Relative to the baseline SCA, the future mean monthly SCA decreases significantly in all warming scenarios and GCMs. Also, relative to the baseline, the period of snow coverage will be reduced. For instance, the ECE3 based SSP5-end-century scenario indicates no snow in the basin in July and August. The SCA differences from the baseline period to the future are minimum for winter and maximum for summer months. These trends are consistent for both GCMs with slightly higher SCA in the ESM based simulations. The changes in SWE from baseline indicated the winter months would have more SWE relative to the baseline period. However, the mean monthly SWE (Table 4) will differ significantly in both GCMs.

**Table 3:**
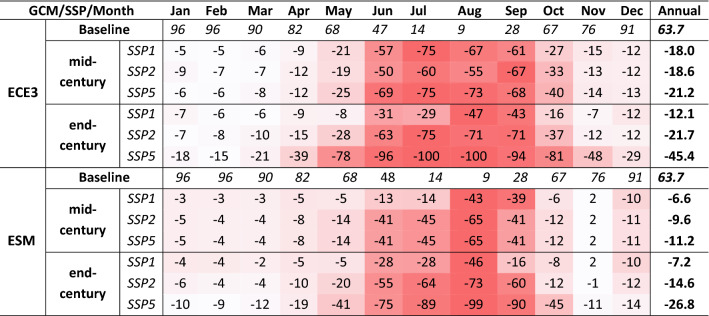
Percentage changes in mean monthly future SCA relative to the baseline for all scenarios based on both GCMs.

**Table 4:**
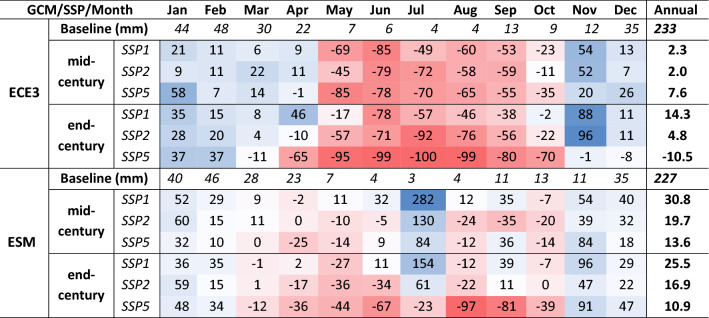
Percentage changes in mean monthly future SWE relative to the baseline for all scenarios based on both GCMs.

## Discussion

Temperature is one of the most important CC indicators with a high impact on the basin’s hydrology. The future basin temperature increases for all months but is highest in summer. The increase severely affects a highly glaciated and snow-fed basin like the Hunza. So, the Hunza basin could be very sensitive to temperature as it controls glacier- and snow melt. Moreover, an increased temperature will significantly affect the precipitation dynamics as an increase will cause more precipitation as rain. This will ultimately change the hydrological dynamics by increasing the flow due to liquid precipitation, and less snow will be stored to contribute during the melt season. Lutz et al.^30^ concluded that UIB had warming between + 2.1 to + 8.0 °C from 1971 to 2000 based on representative concentration pathway (RCP) 4.5 and 8.5 scenarios of CMIP5 projections. The global average indicated a warming of + 1.8 to + 4.4 °C from 1986 to 2005 based on RCP’s scenarios of CMIP5 projections^31^. Similar strong summer warmings for the Indus basin are suggested by Ali et al.^32^. Although the different climate models with different scenarios used in previous studies are difficult to compare, the current study also indicated the warming between + 1.1 and + 8.6 °C by the end of the twenty-first century, depending on the scenarios and GCMs used.

The spatial precipitation pattern for the Hunza basin (Fig. 3) indicated that the northeast parts are receiving less precipitation than the southwest of the basin for both; baseline and future periods. These trends are similar for both GCMs and consistent for all scenarios. The Naltar station recorded the maximum precipitation (annual mean of 718 mm from 1997 to 2010 data). This station is located in the southwest part of the basin. Similarly, the Khunjrab station recorded minimum precipitation (annual mean of 206 mm from 1997 to 2010 data), and this station is located in the northern part of the basin. So, the spatial estimates by GCMs baseline data with more precipitation in the south part of the basin and less in the North part are consistent with the station data. The elevation-distributed GCMs baseline precipitation shows a negative gradient for zone 1–9 and a positive gradient for zone 9–10. The station data showed a similar negative precipitation gradient in the Hunza Basin. The lowest elevation station in the basin (Naltar, 2810 masl) recorded its maximum annual precipitation of 832 mm for 2000 (an average of 701 mm from 1998 to 2010). The middle elevation station (Ziarat, 3669 masl) recorded its maximum of 578 mm in 2004 (an average of 242 mm 1998–2010), and the highest station (Khunjrab, 4730 masl) recorded its maximum of 335 mm in 2010 (an average of 190 mm from 2003 to 2010). So, the pattern of more precipitation at lower elevations by the GCMs baseline is consistent with the observed data. Dahri et al.^3^ also found increased future precipitation in the Karakoram region, where the Hunza basin is located. Su et al.^33^ found an increase in future annual and summer temperatures and monsoon precipitation for this region. However, the large variability in quantitative estimates and spatio-temporal distribution of the projected precipitation is evident in various GCM outputs^7^. Dahri et al.^3^ concluded that no GCM could precisely capture the influence of predominant weather systems. Consequently, significant biases are evident in GCM’s precipitation estimates.

The glacier melt contribution for the baseline period is slightly higher using ESM based inputs than ECE3. However, ECE3 based simulations show slightly more melt contribution for future periods. This discrepancy is associated with a slightly higher baseline temperature in ESM than in ECE3. In contrast, the future temperature in ECE3 is slightly higher than in ESM. For simulations based on future projections, glaciers at all elevations are melting significantly and contributing to the flow. Relative to the baseline temperature, higher elevations generate more melt due to increased temperature at these elevations. The glacier melt is associated with two main drivers; the energy input and the glacier coverage. The energy inputs are related to the temperature, so a higher temperature means more energy available for melt. The fraction of glacier extent present in each elevation is another primary driver that controls melt. With about 31% of glacier extent, nearly 50% of the flow is from glaciers. With a temperature increase in the future, the increased melting will increase river flow. This trend may continue for a few years or decades until the glacier coverage declines sufficiently. When this happens, water contribution from glacier melt is reduced along with the flow. However, the estimation of the future glacier area change is beyond the scope of the current study.

Hasson^5^, Lutz et al.^34^ suggested a similar enhanced glacier melt contribution and increased water availability until around the mid- twenty-first century. An increased future water availability from the Hunza, Astore and Gilgit sub-basins of the UIB under a scenario of the intact glacier is also suggested by Hasson^5^. Tahir et al.^20^ suggested a twofold water availability in the future from the Hunza sub-basin in response to the hypothetical warmer climates till the end of the twenty-first century. Bocchiola et al.^35^ also suggested a consistent increase in water availability in the mid of twenty-first century for the Shigar (a Karakorum based sub-basin of the Indus) due to enhanced glacier melt until the glacial extent reduces to 50%. Hasson^5^ suggested that the warmer climate in the far-future scenario (2087–2097) would increase glacier melt and overall water availability. Soncini et al.^36^ reported negligible ice cover changes under warmer climates projected under various RCP scenarios by the mid-century. Hewitt^37^, Hewitt^38^ and Quincey et al.^18^ reported that the Pamir and Karakoram glaciers have neutral mass balances with even advancing glaciers. Sharif et al.^17^ and Tahir et al.^20^ also indicated that these glaciers are not yet experiencing accelerated melt, possibly due to the Karakoram anomaly^18^. However, this explanation is still hypothetical and requires further investigation and interpretation of the atmospheric dynamics of high-altitude precipitation^39^. Consequently, there is huge uncertainty regarding future glacier extent. The current study, however, presents more realistic future basin-scale and elevation-distributed GM simulations. The highest elevation (a10) has the maximum glacier extent (about 27% of the total) in the Hunza basin. In the baseline period simulations, this elevation zone has an insignificant contribution (1–2%) to the total melt. However, this contribution becomes 16–22% for future simulations. About 68% of the glaciers are located in the upper half of the Hunza basin so there will be a substantial increase in GM from high elevations in the Hunza basin.

Future glacier extent scenarios are crucial for deriving and understanding the future hydrological regime. With about 31% glacier area [RGI, V6.0^25^] of the Hunza basin’s total area, glaciers significantly impact the basin’s hydrological regime. Glacier extent could surely be different in future, but recession scenarios are difficult to validate. Glacier extent was considered constant in this study, but the changed area of glaciers should, however, be considered in future studies.

The simulated flow and hydrograph for the baseline period and their comparison with the observed flow (Fig. 1b,c) show the DDD model’s capacity to reproduce the hydrological dynamics. The model was used to bias correct the GCM data, and validation results suggest a successful application of the model for such a task. Evaluating the bias-corrected CMIP6 GCMs baseline precipitation and temperature data indicates that these products can inform the prevailing hydro-climatic dynamics for river basins such as the Hunza. The Hunza basin is located in the westerlies influenced region, where most of the precipitation falls as snow in winter. Simulations also suggest the Hunza river’s flow is mainly based on meltwater from snow and glaciers.

The short-term peaks in the observed and baseline (simulated) flow are primarily associated with variations in air temperature and energy inputs. The high flow regime of the Hunza river (April–Sep) is controlled by the melt processes, which are primarily associated with temperature and energy variations. Moreover, the snow and temperature inputs, snow spatial distribution, and limitations in the model’s structure may cause the flow discrepancy. Tahir et al.^20^ underestimated the peak flows for the Hunza Basin and suggested that the precipitation input is responsible. Shrestha et al.^40^ also observed the discrepancies in simulated and observed flow peaks for the Hunza river and associated them with input data. Lutz et al.^7^ also underestimated the flood peaks for the Hunza Basin, and they associated them with the temperature input. The increased future flow relative to the baseline (Fig. 4) is mainly associated with increased precipitation and glacier melt. Depending on the scenarios and GCMs used, the glacier melt contribution to the future flow shows an increase between 38–218%, and precipitation shows an increase between 13–58%. The increased temperature will accelerate the melting process, and the stored snow will melt earlier. Moreover, precipitation as rainfall will be more frequent than snowfall due to the projected temperature increase.

**Figure 5:**
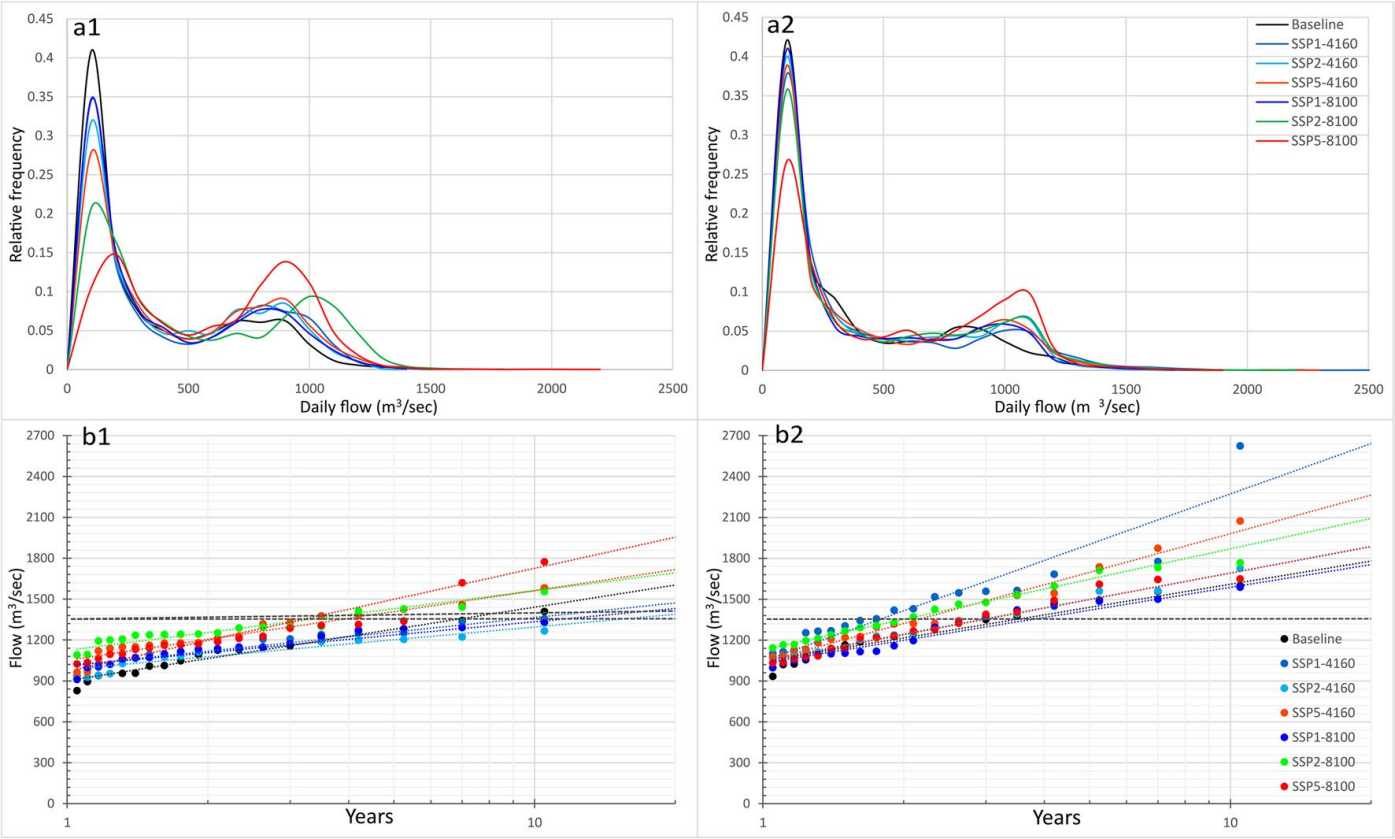
Normalised frequency diagram of baseline and future simulated flow based on; (**a1**) ECE3 and (**a2**) ESM GCM, and annual exceedance of flow under all future CC scenarios; (**b1**) ECE3 and (**b2**) ESM GCM.

Figure 5a1,a2 shows the relative frequency plots of the baseline and future simulated daily flow for both GCMs under all selected scenarios. The daily flow using the baseline and future projections reveal a bimodal probability distribution. The first peak frequency densities correspond to the low flow regime and the second peak densities to the high flow regime (described in the observed data section). Relative to the baseline period flow, the future flow indicates the decreased frequency of the low flow due to the reduced period of flow regime. The increased frequency of high flow with an extended period is associated with the increased glacier melt and precipitation contribution. The Hunza’s mean flow is expected to increase between 39–93% for mid-century and 31–126% for end-century ECE3 based simulations, relative to the baseline period’s mean flow. For ESM based simulations, this future flow is expected to increase between 30–62% for mid-century and 38–172% for end-century.

Based on the future projected trends in precipitation and melts, high flow conditions are expected to occur more frequently in the Hunza basin. Hunza river witnessed very severe flooding in 1994 and 2010. WAPDA flow data from 1991–2010 indicates these years had daily peak flow above 1500 m^3^/s during the flooding period. So, if the flow is higher than 1500 m^3^/s, there could be flooding in the river. Figure 5b1,b2 shows the annual flow exceedance for different return periods under different warming scenarios projected for; (a) ECE3 and (b) ESM GCM. The ESM based daily flow simulations show more occurrences of flow exceeding 1500 m^3^/s than ECE3 based simulations during the twenty-first century for Hunza. The highest mean flow is simulated for ECE3 based SSP5-end-century scenario, with a projected increase of 126% relative to the baseline period flow.

The economy of the Indus region largely depends upon irrigated agriculture controlled through Indus Basin Irrigation System (IBIS). The IBIS is the largest worldwide, irrigating 17 million hectares (M ha) of 24 M ha of the cultivable area in Pakistan^16,41^. The water for the IBIS is dependent on meltwater originating from the HKH region. This water is regulated by two major reservoirs, i.e. Tarbela on River Indus and Mangla on River Jhelum^16^. There is increasing food demand in line with remarkable population growth in the Indo-Gangetic plain. Increased projected river flow and more frequent floods will influence the downstream water availability and management. These significant changes in future flow regimes will severely affect vulnerable communities in the valleys and plains of IBIS. Hence the water resources management in the basin will require serious efforts and strategies regarding hydropower production, reservoir operation, irrigation withdrawals, flood control, and drought management. This can result in increased agricultural productivity and improved livelihoods of the downstream rural communities.

The current study uses two relatively fine resolution GCM projections to evaluate the future hydro-climatic regime of the Hunza basin. Significant differences in the future projected river flows within the same scenario are mainly due to differences in the GCM projected future climates (precipitation and temperature).

This study has several limitations associated with input data and model structure. The elevation-distributed precipitation was derived using GCM data with relatively low spatial resolutions to accurately represent spatial variability in the basin. Also, the bias correction of the GCM data is carried out using the ERA5-Land data as the best available alternative to observations. The flow simulations based on these bias-corrected GCM projections are reasonable. However, with more than 3000 glaciers in UIB, no observed data is available to assess the glacier melt contribution to the flow^6^. Another uncertainty comes from distinguishing between debris-covered and debris-free glaciers^16^. The melt rate for debris-covered glaciers differs from debris-free glaciers^42^, leading to uncertainty in glacier melt simulation. The DDD hydrological model used in the current study is validated and applied with newly developed fine resolution precipitation datasets. The sub-routines for the simplified energy balance approach estimating snowmelt, glacier melt and evapotranspiration have shown promising results. Yet, the model slightly overestimates the SCA. With the development and availability of more fine resolution GCMs, a more comprehensive study using multiple GCM ensembles can be done in the future.

## Conclusions

Pakistan is among the water-scarce countries, and its water resources are highly vulnerable to CC. This study analysed the possible impact of CC on future water availability in the Hunza river basin of the UIB. Novel and relatively fine resolution precipitation and temperature projections were bias-corrected and used by a recently developed hydrological model. The current and future (mid-century (2041–2060) and end-century (2081–2100)) hydrological regimes are simulated using CC scenarios based on GCMs from the recent CMIP6. The following conclusions can be drawn from the current study;

- Increasing temperature is evident for all future CC scenarios, with a basin-scale increase between 1.1 and 8.6 °C. This temperature increase will have significant and even severe implications on a snow- and glacial melt dependent river basin like the Hunza.
- Increasing trends in precipitation are evident in the future period under all warming scenarios. Relative to the baseline period, the ECE3 GCM shows 19–32% increases in annual precipitation, and ESM shows 12–28% increases for the twenty-first century. Moreover, changes in precipitation cycles and their timings are expected, with a reduction in precipitation as snow and an increase in precipitation as rainfall.
- The study presents more realistic future elevation-distributed GM simulations. With the current glacier extent, almost 50% of the annual flow comes from glacier melt. Relative to the annual glacier melt of 2193 Mm^3^ for the baseline period, the simulations show a melt volume increase between 3027 and 5813 Mm^3^ (38–265%) from the Hunza basin by the end of the twenty-first century. The elevation-distributed glacier melts simulations suggest an increasing glacier melt contribution from all elevations with a significant increase from the higher elevations because about 68% of the glaciers are located in the upper half of the Hunza basin. Such a substantial increase in glacier melt can significantly change Hunza’s flow regime, which can be alarming.
- Relative to the baseline period flow, the low flow regime is expected to have increased flow with the flow period reduced to a few months. Also, for the high flow regime, the flow is expected to increase with the flow period expanding from July-Sep to May–October. This increased frequency of high flow with an extended period is associated with increased precipitation and glacier melt contributions.
- The future flow varies highly under different warming and GCM projections. Overall increasing trends in the future river flow projections are evident, with the projected increase between 23 and 126% relative to the baseline flow, depending on the scenarios and GCMs used.
- High flow conditions with more frequent floods are expected in the Hunza basin. Relative to the peak flood of ~ 1600 m^3^/s during the baseline period, flood magnitude can be as high as ~ 2800 m^3^/s in the future period. In addition, high flow frequencies are expected to increase in future periods based on all the scenarios and GCMs used. These floods can severely impact vulnerable communities in the narrow valleys and downstream plains. Moreover, increased river flow will influence the downstream water availability and management.

The increased flow and changes in the flow seasonality due to increased precipitation and glacier melt will significantly affect the hydrological regime. These changes in flow regimes could adversely or positively affect agricultural production and ecology. Moreover, an increased population combined with increased energy and food demands will mean more demand on water resources. The findings improve understanding of the future hydro-climatic regime by providing helpful information about the meltwater contributions and hydrological regimes. The future flow regime of the Hunza presented in the current study will be informative for the larger region. Finally, the findings in this study may assist relevant stakeholders and policymakers regarding hydropower and reservoir development, sustained agriculture production, CC adaptation, and efficient management of water resources.

## Methods

### Study area

**Figure 6:**
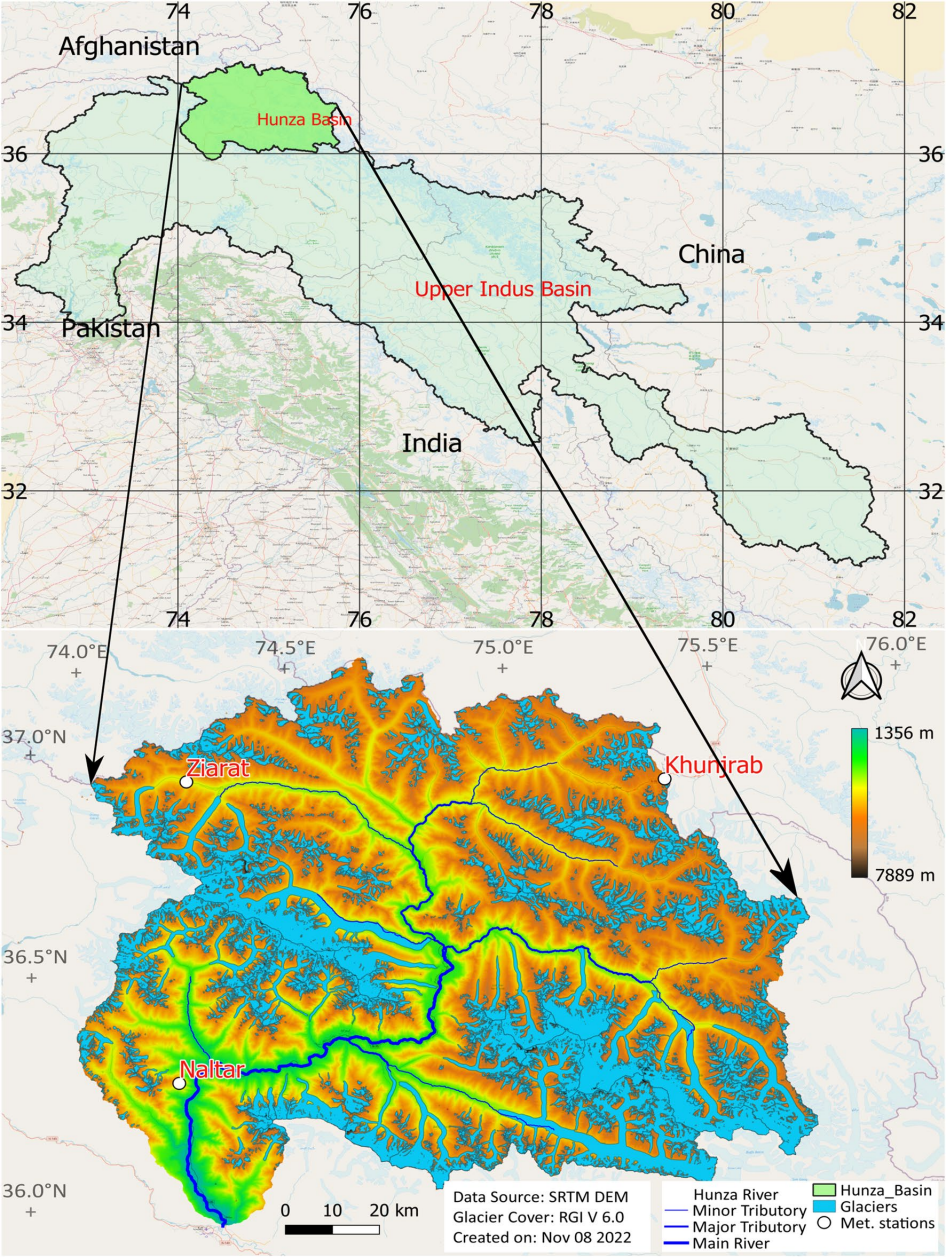
Location of the study area, glacier extent, river network and meteorological stations.

The Hunza Basin, with an area of 13,713 km^2^, lies in the western Karakoram mountains of the HKH region. The basin stretches between 74.04–75.77° E and 36.05–37.08° N. Figure 6 shows the location, digital elevation model (DEM), drainage area, meteorological stations, and glacier coverage of the basin. The basin has a complex topography with deep valleys and extreme topographic relief with elevations between 1456 and 7822 m above sea level (masl) and a mean elevation of 4600 masl. The Hunza is one of the main sub-basins of the Upper Indus Basin (UIB), and it contributes about 12% of the total flow of UIB upstream of the Tarbela reservoir^40^. The Hunza is a snow-fed and highly glaciated basin. Seasonal snow is at its maximum in winter, with almost 85% of its total area covered with snow^40^. The basin has a glacier extent of about 31% of the total area and is located between 2300 and 7889 masl (RGI V6.0^25^) (Table S4). The basin hosts some extensive glacier systems, including Hispar (339 km^2^), Batura (238 km^2^), Virjerab (112 km^2^), Khurdopin (111 km^2^) and a few others. The basin has a dense river network with Hunza as the main tributary (232 km long). The 1966–2010 Hunza river flow data collected by Pakistan’s Water and Power development authority (WAPDA) showed an average flow of 304 m^3^/s (~ 710 mm). The climate in the Hunza basin is arid to semiarid and is generally divided into four seasons; winter (Dec–Feb), spring (March–May), monsoon (June–Sep), and post-monsoon season (Oct–Nov)^43^. The HKH precipitation has two primary sources; summer monsoons and winter westerlies. The Hunza basin receives precipitation from both sources, with about two-thirds from the winter westerlies and one-third from the summer monsoon^44^. Precipitation at Hunza peaks around March/April under the westerlies regime, followed by August/September under the monsoon^5^.

### Observed data

#### Hydrometeorological data

The observed hydrometeorological data are obtained from WAPDA and the Pakistan Meteorological Department (PMD). Observed temperature data are used to derive the elevation-distributed temperature and bias-correct the GCM based temperature projections for the Hunza basin. The observed precipitation data are not used as input to the model but to compare the spatial and seasonal precipitation estimates by ERA5-Land. The Hunza basin has three meteorological stations (Naltar 2810 masl, Ziarat 3669 masl, and Khunjrab 4730 masl). From 1997–2010, the Naltar and Ziarat stations recorded monthly maximum precipitation in April and minimum in November. The Khunjrab station recorded monthly maximum precipitation in August and minimum in October. The Naltar station recorded maximum annual precipitation of 701 mm, and the Khunjrab station recorded a minimum of 190 mm. The annual average temperature is 6.6 °C, 3.0 °C and − 5.01 °C at Naltar, Ziarat and Khunjrab stations. The monthly mean temperature is maximum in July and minimum in January at all stations. The flow gauge of the Hunza basin is installed at Danyore Bridge (1356 masl). The flow of the Hunza River shows two major flow regimes. One is the low flow regime (October–March), and the second is the high flow regime (April–September). The high flow regime is further divided into snowmelt dominated (April–mid June) and glacier-melt dominated (mid June–September)^5^. The observed flow is used for the DDD model calibration and validation for observed data based simulations (1997–2010). The flow data are also used to validate the GCM based simulation of daily flow for the baseline period (1991–2010).

#### ERA5-Land

The UIB is a data-scarce basin with very few installed stations for climatic data. These stations have a limited record period, and the records contain missing data. The high altitude, altitudinal variations and complex weather systems complicate the monitoring^43,45,46^. Hence, the existing stations inadequately capture the precipitation amounts and patterns. Precipitation estimates from the ERA5-Land gridded dataset were reasonable for the UIB^26,43,47^. The ERA5-Land data are newly developed, available from 1981 to the near real-time with several weeks delay with an hourly temporal resolution, 0.1° × 0.1° spatial resolution and with a global spatial coverage^48^. The dataset is freely available at https://cds.climate.copernicus.eu/ and was accessed in January 2021. The ERA5-Land data are used to derive elevation-distributed precipitation for the Hunza basin from 1997 to 2010.

#### APHRODITE

The Asian Precipitation-Highly Resolved Observational Data Integration Towards Evaluation of water resources (APHRODITE) is a temperature and precipitation dataset. The dataset is developed from a network of gauges in Asia and is available from 1951 to 2015. The dataset has a daily temporal resolution, a 0.25° × 0.25° spatial resolution and spatial coverage of 60–150° E, 15–55° N. These data are based on a gauge network and an improved interpolation algorithm where the local topography between the gauges and interpolated point is considered^49^. The dataset is freely available at http://www.chikyu.ac.jp/precip/ and was accessed in January 2021. The APHRODITE temperature data are used to derive the temperature lapse rate for the higher elevation of the Hunza basin, where no station/reference data are available.

#### CMIP6 GCM data

With the availability of a large number of GCM outputs, the spread and variability in their outcomes are also large^30^. Considering these uncertainties, one approach would be using all available models; however, CMIP6 dynamically downscaled (i.e. fine resolution) data is not available yet. Furthermore, for a relatively small catchment with a huge altitudinal variation like the Hunza, a coarse resolution GCMs cannot accurately represent the spatial variability. Considering these limitations, ECE3 and MPI-ESM GCMs were selected based on their spatial resolution in this study. Also, compared with station observations of precipitation and temperature, these GCMs performed better for overall quantification and showed smaller biases. The GCMs dataset was used to derive the Hunza basin’s temperature and precipitation for the baseline (1991–2010) and future (2041–2060 and 2081–2100) periods under the Shared Socioeconomic Pathways (SSP) scenarios of SSP1, SSP2 and SSP5.

#### EC-Earth3

CMIP6 based EC-Earth is a state-of-the-art European community Earth-System model (ESMs) developed by the European EC-Earth consortium, including about 20 institutions^50^. ECE3 is a GCM developed in a collaborative and decentralised way^51^. The ECE3 used in CMIP6 has daily and sub-daily temporal resolution, a spatial resolution of 0.7° × 0.7° and global spatial coverage. The dataset is freely available at https://esgf-node.llnl.gov/projects/cmip6/ and was accessed in October 2021.

#### MPI-ESM

MPI-ESM is the Max Planck Institute’s Earth System Model (MPI-ESM1.2)^52^. The MPI-ESM for CMIP6 has daily and sub-daily temporal resolution, a 0.9° × 0.9° spatial resolution and global spatial coverage. MPI-ESM is freely available at https://esgf-node.llnl.gov/projects/cmip6/ and was accessed in October 2021.

### Satellite data

The satellite datasets used in the current study include the Shuttle Radar Topography Mission (SRTM) DEM, the Randolph Glacier Inventory version 6.0 (RGI V6), the Landsat-8 and the Moderate Resolution Imaging Spectroradiometer (MODIS). The SRTM DEM dataset was developed by the United States National Aeronautics and Space Administration (NASA) in 2013 with 30 m spatial resolution. The DEM data were used for catchment delineation, hypsometry and river network (Fig. 6). The RGI dataset was developed by the Global Land Ice Measurement from Space (GLIMS) in 2017 with a 30 m spatial resolution. The dataset was developed to monitor the glacier extent globally. The RGI data were used to derive the elevation-distributed glacier extent in the Hunza basin (Fig. 6, Table S4). Landsat-8 data are developed by the Landsat Data Continuity Mission (LDCM) with 30 m spatial and 16 days temporal resolution. The Landsat-8 data were used to derive the land cover and the distance distributions from the bogs and hillslopes to the nearest stream used as DDD model parameters^53^. The MODIS snow data were used to validate the model’s SCA simulations and were accessed from^54^ for the Hunza basin. The DEM, RGI and Landsat-8 are freely available and acquired from their official websites.

### Modelling framework

#### DDD model

The DDD model developed by Skaugen and Onof^55^ of the Norwegian Water Resources and Energy Directorate (NVE) is a semi-distributed, precipitation-runoff model written in the programming languages of R and Julia. The model simulates river flow, the elevation-distributed SCA, SWE, GM, potential and actual evapotranspiration (E.P. and E.A.) and subsurface water storage^53^. The model is data and parameter parsimonious and only needs elevation-distributed precipitation and temperature as input. In the current modelling setup, the model’s energy balance based sub-routines are employed to calculate the evapotranspiration, snow- and glacial melt. Further details on the model’s description and setup can be found in Skaugen and Onof^55^ and Nazeer et al.^43^. Table S1 shows the DDD model’s calibration parameters with the calibration range and values used in the current simulations. Table S2 shows the DDD model’s GIS derived parameters and parameters with fixed values.

#### Bias correction of GCM projections

The GCM projections are subjected to various uncertainties and model biases. Therefore, these GCM projections require bias correction before being applied for future climatic and hydrological investigations^3^. Previous studies^5,45,46,56^ evaluated the performance of different bias correction techniques. The station data can be helpful for bias correction, but as mentioned previously, the Hunza basin is data-scarce. So the global precipitation data of ERA5-Land, with its latest release and fine resolution^3,43,47^, were used for GCM bias-correction in this study. The ERA5-Land precipitation data, however, overestimate the precipitation and need to be corrected before being applied as the observed precipitation data for the Hunza basin. The GCM baseline projections are then corrected using the corrected ERA5-Land precipitation; the same corrections are then applied to GCM’s future projections. Hence, the precipitation bias correction consists of three steps, discussed in the following sections.

#### Model setup using ERA5-Land

The DDD model is run using daily scale precipitation and temperature as input. The precipitation is derived from the ERA5-Land, and temperature from station data, with a temperature lapse rate derived from station data and APHRODITE temperature data^26^. Calibrating against the observed flow, the DDD model suggests a precipitation correction factor, separately for rainfall and snow. To do so, the model decides if the precipitation is rainfall or snow using a calibrated temperature threshold (TX) and calibrates the correction factors for rain (Pkorr) and snow (Skorr) separately. The DDD model was calibrated from 1997 to 2005 and validated from 2006 to 2010. The elevation-distributed correction factors for rain and snow are used to bias-correct the GCM’s elevation-distributed projections.

#### Bias correction of GCM projections

The selected GCMs’ precipitation and temperature are bias-corrected using the mean-based method^57,58^. The correction of precipitation projections is based on “observed precipitation” (corrected ERA5-Land data), and the correction of temperature projections is based on “observed temperature” (station data and APHRODITE lapse rate). The methods adopted for temperature and precipitation bias correction are shown in Eqs. (1) and (2), respectively.

1 $$T_{M} \prime (i) \, = T_{M} (i) \, + \mu_{O} - \mu_{M}$$

2 $$P_{M} \prime \, (i) \, = P_{M} (i) \, \times \mu_{O} \prime /\mu_{M} \prime$$

where $T_{M} \prime$ is the bias-corrected daily temperature, *T*_*M*_ is the daily model (GCM) temperature before bias correction, *i* is a day in the month, and μ_*O*_ and μ_*M*_ are monthly means of observed and model (GCM) temperature for the baseline period, respectively.

$P_{M} \prime$ is the bias-corrected daily precipitation, *P*_*M*_ is the daily model (GCM) precipitation before bias correction, *i* is a day in the month, and $\mu_{O} \prime$ and $\mu_{M} \prime$ are monthly means of observed and model (GCM) precipitation for the baseline period, respectively.

For the future periods (2041–2060 (mid-century) and 2081–2100 (end-century)), $\mu_{O} \prime /\mu_{M} \prime$ for precipitation and μ_*O*_–μ_*M*_ for temperature are taken from the baseline period (1991–2010) at a monthly basis.

#### Model setup using GCM projections

When running the DDD model with GCMs projections, the calibrated parameters are unchanged except for the precipitation correction factors. These correction factors are kept at ‘1’ since the elevation-distributed precipitation is already bias-corrected. DDD is run for GCMs for the baseline (1991–2010), mid-century (2041–2060) and end-century (2081–2100) period.

### Performance analysis

Calibration and validation of the DDD model are performed for corrected ERA5-Land precipitation and observed/extrapolated temperature on a daily time step from 1997–2005 and 2006–2010, respectively. The efficiency criteria of KGE and NSE are used for accuracy assessment and evaluation. The KGE (Eq. 3) is a goodness-of-fit measure developed by Gupta et al.^59^. KGE is increasingly being used for model evaluation and has values ranging from minus infinity to one. The NSE (Eq. 4)^60^ assesses the relative magnitude of residual variance compared with the variance in measured data subtracted from unity.

3 $$KGE=1-\sqrt{{\left(r-1\right)}^{2}+{\left(\frac{\sigma sim}{\sigma obs}-1\right)}^{2}+{\left(\frac{\mu sim}{\mu obs}-1\right)}^{2}}$$

4 $$NSE=1- \frac{\sum_{i=1}^{n}{({Qobs}_{i}- {Qsim}_{i})}^{2}}{\sum_{i=1}^{n}{({Qobs}_{i}- \overline{Qobs })}^{2}}$$

where; r is the linear correlation between simulations and observations, $\sigma {\text{sim}}$ and $\sigma \text{obs}$ are the standard deviations of simulations and observations, $\mu \text{sim}$ and $\mu \text{obs}$ are the means of simulations and observation, $Qobs$ is the observed flow and $Qsim$ is the simulated flow, for day i, $\overline{Qobs }$ is the mean observed flow over the n number of days.

## Supplementary Information

Supplementary Information.

## Data Availability

The datasets used for the current study are available either as published literature or as open source data. We have cited the source for accessing the data at appropriate places. The results generated during the current study are available either in the paper and/or supplementary data or can be obtained from the corresponding author upon request.

## References

[1] Masson-Delmotte, V., Zhai, P., Pirani, A., Connors, S. L., Péan, C., Berger, S., Caud, N., Chen, Y., Goldfarb, L., Gomis, M. I., Huang, M., Leitzell, K., Lonnoy, E., Matthews, J. B. R., Maycock, T. K., Waterfield, T., Yelekçi, O., Yu, R. & Zhou, B. (eds.) IPCC, 2021: Climate Change 2021: The Physical Science Basis. Contribution of Working Group I to the Sixth Assessment Report of the Intergovernmental Panel on Climate Change (2021).

[2] Ciais P (2014). Climate Change 2013: The Physical Science Basis. Contribution of Working Group I to the Fifth Assessment Report of the Intergovernmental Panel on Climate Change.

[3] Dahri ZH (2021). Climate change and hydrological regime of the high-altitude Indus basin under extreme climate scenarios. Sci. Total Environ..

[4] League, E. *et al.* United in science: High-level synthesis report of latest climate science information convened by the science advisory group of the UN climate action summit 2019 (2019).

[5] Hasson SU (2016). Future water availability from Hindukush-Karakoram-Himalaya upper Indus Basin under conflicting climate change scenarios. Climate.

[6] Hasson SU, Saeed F, Böhner J, Schleussner C-F (2019). Water availability in Pakistan from Hindukush–Karakoram–Himalayan watersheds at 1.5° C and 2° C Paris Agreement targets. Adv. Water Resour..

[7] Lutz AF, Immerzeel WW, Kraaijenbrink PD, Shrestha AB, Bierkens MF (2016). Climate change impacts on the upper Indus hydrology: Sources, shifts and extremes. PLoS One.

[8] Stocker, T. F. *et al.* (Cambridge University Press, 2013). Advanced concepts on remote sensing of precipitation at multiple scales.

[9] Barnett TP, Adam JC, Lettenmaier DP (2005). Potential impacts of a warming climate on water availability in snow-dominated regions. Nature.

[10] Wijngaard RR (2018). Climate change vs. socio-economic development: Understanding the future South Asian water gap. Hydrol. Earth Syst. Sci..

[11] Cannon F, Carvalho LM, Jones C, Bookhagen B (2015). Multi-annual variations in winter westerly disturbance activity affecting the Himalaya. Clim. Dyn..

[12] Archer DR, Fowler HJ (2004). Spatial and temporal variations in precipitation in the Upper Indus Basin, global teleconnections and hydrological implications. Hydrol. Earth Syst. Sci..

[13] Lalande M, Ménégoz M, Krinner G, Naegeli K, Wunderle S (2021). Climate change in the High Mountain Asia in CMIP6. Earth Syst. Dyn..

[14] Abbas A (2022). Evaluation and projection of precipitation in Pakistan using the Coupled Model Intercomparison Project Phase 6 model simulations. nt. J. Climatol..

[15] Shafeeque M, Luo Y (2021). A multi-perspective approach for selecting CMIP6 scenarios to project climate change impacts on glacio-hydrology with a case study in Upper Indus river basin. J. Hydrol..

[16] Akhtar M, Ahmad N, Booij MJ (2008). The impact of climate change on the water resources of Hindukush–Karakorum–Himalaya region under different glacier coverage scenarios. J. Hydrol..

[17] Sharif M, Archer D, Fowler H, Forsythe N (2013). Trends in timing and magnitude of flow in the Upper Indus Basin. Hydrol. Earth Syst. Sci..

[18] Quincey D (2011). Karakoram glacier surge dynamics. Geophys. Res. Lett..

[19] Hewitt K (2005). The Karakoram anomaly? Glacier expansion and the ‘elevation effect’, Karakoram Himalaya. Mt. Res. Dev..

[20] Tahir AA, Chevallier P, Arnaud Y, Neppel L, Ahmad B (2011). Modeling snowmelt-runoff under climate scenarios in the Hunza River basin, Karakoram Range, Northern Pakistan. J. Hydrol..

[21] Mukhopadhyay B, Khan A (2014). Rising river flows and glacial mass balance in central Karakoram. J. Hydrol..

[22] Rees, G. & Collins, D. An assessment of the potential impacts of deglaciation on the water resources of the Himalaya. *Snow and glacier aspects of water resources management in the Himalayas, Centre for Ecology and Hydrology, Oxfordshire, UK, Technical Reports, DFIP KAR Project* (2004).

[23] Bernstein, L. *et al.* (IPCC, 2008). IPCC, 2007: Climate Change 2007: Synthesis Report.

[24] Mukhopadhyay B, Khan A, Gautam R (2015). Rising and falling river flows: Contrasting signals of climate change and glacier mass balance from the eastern and western Karakoram. Hydrol. Sci. J..

[25] Arendt, A. *et al.* Randolph Glacier Inventory—A Dataset of Global Glacier Outlines: Version 6.0: Technical Report, Global Land Ice Measurements from Space (2017).

[26] Nazeer, A., Maskey, S., Skaugen, T. & McClain, M. E. Analysing the elevation-distributed hydrological regime of the highly glaciated and snow-fed Hunza Basin in the Hindukush Karakoram Himalaya (HKH) region. *J. Hydrol. Reg. Stud. *(2022) **(under review)**.

[27] Klok E, Jasper K, Roelofsma K, Gurtz J, Badoux A (2001). Distributed hydrological modelling of a heavily glaciated Alpine river basin. Hydrol. Sci. J..

[28] Eyring, V. *et al.* Overview of the Coupled Model Intercomparison Project Phase 6 (CMIP6) experimental design and organisation. *Geosci. Model Dev. Discuss.* **8** (2015).

[29] Gidden MJ (2019). Global emissions pathways under different socioeconomic scenarios for use in CMIP6: A dataset of harmonized emissions trajectories through the end of the century. Geosci. Model Dev..

[30] Lutz A (2016). Selection of Climate Models for Developing Representative Climate Projections for the Hindu Kush Himalayan Region.

[31] Knutti R, Sedláček J (2013). Robustness and uncertainties in the new CMIP5 climate model projections. Nat. Clim. Change.

[32] Ali S, Reboita MS, Kiani RS (2021). 21st century precipitation and monsoonal shift over Pakistan and Upper Indus Basin (UIB) using high-resolution projections. Sci. Total Environ..

[33] Su B (2016). Statistical downscaling of CMIP5 multi-model ensemble for projected changes of climate in the Indus River Basin. Atmos. Res..

[34] Lutz A, Immerzeel W, Shrestha A, Bierkens M (2014). Consistent increase in High Asia's runoff due to increasing glacier melt and precipitation. Nat. Clim. Change.

[35] Bocchiola D (2011). Prediction of future hydrological regimes in poorly gauged high altitude basins: The case study of the upper Indus, Pakistan. Hydrol. Earth Syst. Sci..

[36] Soncini A (2015). Future hydrological regimes in the upper Indus Basin: A case study from a high-altitude glacierized catchment. J. Hydrometeorol..

[37] Hewitt K (2007). Tributary glacier surges: An exceptional concentration at Panmah Glacier, Karakoram Himalaya. J. Glaciol..

[38] Hewitt K (2011). Glacier change, concentration, and elevation effects in the Karakoram Himalaya, Upper Indus Basin. Mt. Res. Dev..

[39] Cogley JG (2011). Present and future states of Himalaya and Karakoram glaciers. Ann. Glaciol..

[40] Shrestha M (2015). Integrated simulation of snow and glacier melt in water and energy balance-based, distributed hydrological modeling framework at Hunza River Basin of Pakistan Karakoram region. J. Geophys. Res. Atmos..

[41] Khan A, Richards KS, Parker GT, McRobie A, Mukhopadhyay B (2014). How large is the Upper Indus Basin? The pitfalls of auto-delineation using DEMs. J. Hydrol..

[42] Reid T, Carenzo M, Pellicciotti F, Brock B (2012). Including debris cover effects in a distributed model of glacier ablation. J. Geophys. Res. Atmos..

[43] Nazeer A, Maskey S, Skaugen T, McClain ME (2021). Simulating the hydrological regime of the snow fed and glaciarised Gilgit Basin in the Upper Indus using global precipitation products and a data parsimonious precipitation-runoff model. Sci. Total Environ..

[44] Bookhagen B, Burbank DW (2010). Toward a complete Himalayan hydrological budget: Spatiotemporal distribution of snowmelt and rainfall and their impact on river discharge. J. Geophys. Res. Earth Surface.

[45] Dahri ZH (2018). Adjustment of measurement errors to reconcile precipitation distribution in the high-altitude Indus basin. Int. J. Climatol..

[46] Dahri ZH (2016). An appraisal of precipitation distribution in the high-altitude catchments of the Indus basin. Sci. Total Environ..

[47] Syed Z (2022). Hydroclimatology of the Chitral River in the Indus Basin under changing climate. Atmosphere.

[48] Muñoz Sabater J (2019). Copernicus Climate Change Service (C3S).

[49] Yatagai A (2012). APHRODITE: Constructing a long-term daily gridded precipitation dataset for Asia based on a dense network of rain gauges. Bull. Am. Meteorol. Soc..

[50] Hazeleger W (2010). EC-Earth: A seamless earth-system prediction approach in action. Bull. Am. Meteorol. Soc..

[51] Massonnet F (2020). Replicability of the EC-Earth3 Earth system model under a change in computing environment. Geosci. Model Dev..

[52] Gutjahr O (2019). Max Planck Institute Earth System Model (MPI-ESM1.2) for the High-Resolution Model Intercomparison Project (HighResMIP). Geosci. Model Dev..

[53] Skaugen T, Weltzien IH (2016). A model for the spatial distribution of snow water equivalent parameterized from the spatial variability of precipitation. Cryosphere.

[54] Muhammad S, Tian L, Khan A (2019). Early twenty-first century glacier mass losses in the Indus Basin constrained by density assumptions. J. Hydrol..

[55] Skaugen T, Onof C (2014). A rainfall-runoff model parameterized from GIS and runoff data. Hydrol. Process..

[56] Shrestha NK (2017). Evaluating the accuracy of Climate Hazard Group (CHG) satellite rainfall estimates for precipitation based drought monitoring in Koshi basin, Nepal. J. Hydrol. Reg. Stud..

[57] Wang L, Ranasinghe R, Maskey S, van Gelder PM, Vrijling J (2016). Comparison of empirical statistical methods for downscaling daily climate projections from CMIP5 GCMs: A case study of the Huai River Basin, China. Int. J. Climatol..

[58] Sirisena T, Maskey S, Bamunawala J, Ranasinghe R (2021). Climate change and reservoir impacts on 21st-century streamflow and fluvial sediment loads in the Irrawaddy River, Myanmar. Front. Earth Sci..

[59] Gupta HV, Kling H, Yilmaz KK, Martinez GF (2009). Decomposition of the mean squared error and NSE performance criteria: Implications for improving hydrological modelling. J. Hydrol..

[60] Nash JE, Sutcliffe JV (1970). River flow forecasting through conceptual models part I—A discussion of principles. J. Hydrol..

