# Supplementary material for: Changes in the hydro-climatic regime of the Hunza Basin in the Upper Indus under CMIP6 climate change projections
Source: Sci Rep. 2022 Dec 12;12:21442. doi: 10.1038/s41598-022-25673-6 (PMC9744897; doi:10.1038/s41598-022-25673-6)
Supplement: Supplementary file 1 — Supplementary Information. [file 41598_2022_25673_MOESM1_ESM.pdf]

Supplementary Data

Table S1: List of DDD model parameters needs to be calibrated and their values used

Parameters	Description	Unit	Range	Value used
Pcorr	Rain correction factor	fraction	0.40–2.0	*0.60*
Scorr	Snow correction factor	fraction	0.40–2.0	*0.63*
Pro	Liquid content in snow	fraction	0.03–0.1	*0.05*
Tx	Threshold for snow/rain	°C	-2.00–+2.0	*-0.10*
GshInt	Shape parameter	real number	0.5–1.3	*0.69*
GscInt	Scale parameter	real number	0.03–0.05	*0.03*
U	Mean wind speed	m/s	2.0–3.5	*2.02*
Rv	Celerity for river flow	cm/sec	0.50–1.5	*0.62*

Table S2: List of Distance Distribution Dynamics (DDD) hydrological model parameters estimated from Geographic Information System (GIS) and recession analysis

Parameter	Description	Method of Estimation/Source
Hypsographic curve	11 values representing the quantiles starting from 0 to 100	SRTM DEM
Hfelt [m]	Mean altitude of catchment	SRTM DEM
Area [m^2]	Catchment area	SRTM DEM
D	Parameter for spatial distribution of SWE, decorrelation length	From spatial distribution of precipitation
a_0	Parameter for spatial distribution of SWE, shape parameter	From spatial distribution of precipitation
R	Field Capacity	Fixed value of 0.30
maxLbog [m]	Maximum distance distribution for bogs	LandSat-8
midLbog [m]	Mean distance distribution for bogs	LandSat-8
Bogfrac	Bogs fraction in catchment	LandSat-8
MAD [m^3/sec]	Long-term mean annual runoff	From Observed runoff
Zsoil	Zero distance areal fraction for soil in the river network	LandSat-8
Zbog	Zero distance areal fraction for bogs in the river network	LandSat-8
midD [m]	Mean value of distance distribution in hillslope	LandSat-8
maxD [m]	Maximum value of distance distribution in hillslope	LandSat-8
midGL [m]	Mean value of distance distribution of glaciers	LandSat-8
stdGL[m]	Standard deviation for distance distribution of glaciers	RGI 6.0
Glacier fraction	Areal fraction of glaciers in ten elevation zones	RGI 6.0
midFl	Mean distance for river network	SRTM DEM
stdFL	Standard deviation of distance for river network	SRTM DEM
maxFL	Maximum distance for river network	SRTM DEM
NoL	Number of subsurface layers	Fixed value of 5

Table S3: Percentage changes in mean monthly future precipitation relative to the baseline for all scenarios based on both GCMs

GCM/SSP/Month			Jan	Feb	Mar	Apr	May	Jun	Jul	Aug	Sep	Oct	Nov	Dec	Annual
ECE3	***Baseline (mm)***		*44*	*48*	*34*	*30*	*18*	*21*	*30*	*35*	*27*	*9*	*11*	*35*	***343***
	**mid-century**	*SSP1*	18	18	16	-15	74	16	56	30	-11	12	41	6	**19**
		SSP2	8	15	22	5	88	3	28	5	3	26	44	4	**17**
		SSP5	58	9	22	-9	111	11	58	-4	15	38	17	26	**27**
	**end-century**	*SSP1*	35	15	16	23	90	-10	20	12	27	7	74	11	**24**
		SSP2	27	17	18	-11	133	17	13	3	-1	21	89	10	**21**
		SSP5	31	43	8	-26	96	130	59	-14	25	120	-8	-19	**28**
ESM	***Baseline (mm)***		*45*	*52*	*36*	*31*	*17*	*20*	*29*	*33*	*24*	*10*	*12*	*36*	***345***
	**mid-century**	*SSP1*	32	18	19	4	13	-6	59	31	-8	38	43	32	**23**
		SSP2	42	5	13	4	8	-27	43	-29	-45	14	27	25	**9**
		SSP5	15	2	4	-12	24	0	36	9	5	19	66	14	**12**
	**end-century**	*SSP1*	18	25	1	6	-7	-21	13	-30	-10	41	78	23	**10**
		SSP2	40	6	7	-6	23	-16	22	-12	-11	52	37	15	**11**
		SSP5	28	29	-1	-20	80	-32	16	-62	-47	21	61	40	**8**

Table S4: Hypsometry and glacier coverage of the Hunza Basin

Area quantile	Elevation range (masl)	Mean elevation (masl)	Glaciers area (km^2)	Glaciers (%) of total extent
a1	*1425-3217*	*2321*	*67*	*1.6*
a2	*3218-3755*	*3486*	*200*	*4.7*
a3	*3756-4123*	*3939*	*232*	*5.4*
a4	*4124-4403*	*4263*	*255*	*6.0*
a5	*4404-4640*	*4522*	*289*	*6.8*
a6	*4641-4849*	*4745*	*348*	*8.1*
a7	*4850-5053*	*4951*	*437*	*10.2*
a8	*5054-5264*	*5159*	*576*	*13.4*
a9	*5265-5549*	*5407*	*738*	*17.2*
a10	*5550-7889*	*6719*	*1142*	*26.7*

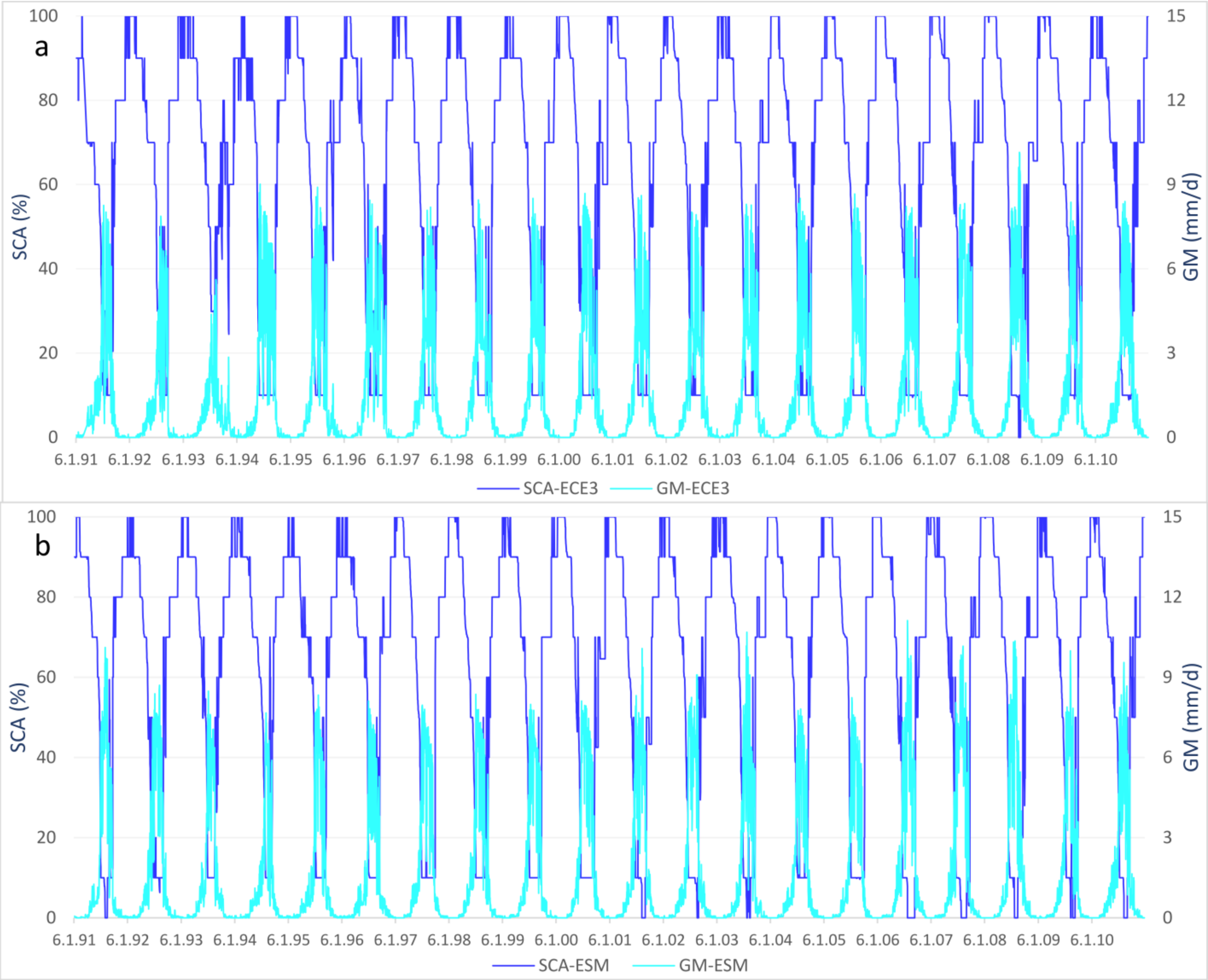

Figure S1: Basin-scale daily simulated snow cover area (SCA) and glacier melt (GM) based on; a) ECE3 and b) ESM GCM
